# Shared Decision Making, Decision Aids and Patient Reported Outcome Measures for Overactive Bladder Care: A Review

**DOI:** 10.1007/s11884-025-00800-x

**Published:** 2026-02-07

**Authors:** Roshan Paudel, Maanasa Bommineni, Giulia M. Ippolito

**Affiliations:** 1 Department of Medical Oncology, Dana-Farber Cancer Institute, Boston, MA 02215 USA; 2 Medical Student, Central Michigan University College of Medicine, Mount Pleasant, MI 48859 USA; 3 Department of Urology, University of Michigan, Ann Arbor, MI 48109 USA

**Keywords:** Shared decision making, Patient preferences and values, Patient reported outcomes, Decision aid, Decision making, Prediction models, Machine learning, Artificial intelligence

## Abstract

**Purpose of Review:**

Shared decision making (SDM) is integral to clinical decision making for OAB. SDM is a collaborative process that takes patients’ values, preferences, and goals into account when deciding on their treatment options. Decision aids (DAs) can support SDM and patient-reported outcomes (PROs) help to assess the outcomes most important to the patient.

**Recent Findings:**

Twenty-five articles were retrieved and reviewed. Our search for literature about SDM in OAB found that physician recommendation is a key decisional component for patients yet that physicians’ priorities may differ widely from patients’ preferences. We evaluated currently available decision aids for OAB and found that none of the peer reviewed aids are publicly available, though non-peer reviewed, paper-based decision aids are available online. At least 10 PROs are available for OAB, these are regularly used in trials of efficacy and are increasingly being implemented in clinical practice. Finally, artificial intelligence applications such as large language models and machine learning based clinical risk prediction tools are emerging as a new facet to augment SDM, but there are limitations on the quality and the clinical implementation of these tools.

**Summary:**

Decision aids and patient reported outcome measures are integral to the delivery of patient-centered, individualized, shared decision making for OAB. Despite this, few freely available DAs exist and many PROs are available, which makes comparison of outcomes between treatments challenging. Emerging AI technologies may further augment the SDM however require validation prior to clinical use.

## Introduction

Overactive bladder (OAB) is a common, chronic syndrome that substantially impacts the health-related quality of life of people experiencing bothersome symptoms. Over a third of women and at least 15% of men equating to nearly 30 million Americans, experience OAB symptoms [1]. These include bothersome urinary urgency and frequency, and urgency associated urinary incontinence [1, 2]. Of those who suffer from OAB, nearly 40% report that the condition interferes with daily life, of which about 25% report that symptoms cause them to avoid leaving the house [1, 2, 9]. Prior qualitative work has detailed the profound social and psychological consequences associated with OAB revealing themes of isolation, social stigma, and feeling invalidated or dismissed [2]. Patients with OAB feel vulnerable and are desperate for a cure [9]. 

Despite its significant impact, decisions for treatment of OAB symptoms remains largely a trial and error approach driven by step-therapy policies and lacking in individual patient-centered approaches [4]. Step-therapy requirements, a tiered treatment pathway used by insurers that mandates documented failure of a prior step before accessing other treatment options, have historically guided the treatment for OAB. This trial and error approach does not take into consideration severity of symptoms, is not patient centered and can be burdensome for patients and clinicians. The 2024 American Urological Association (AUA)/ Society of Urodynamics, Female Pelvic Medicine, & Urogenital Reconstruction (SUFU) Guidelines on Overactive Bladder eliminated the concept of step therapy and instead emphasized the importance of shared decision-making (SDM) between clinicians and patients to select evidence-based therapies that align with patient’s values and preferences [2]. 

In order to practice guideline concordant care for OAB, clinicians must be familiar with SDM and its relationship to Decision Aids (DAs) and patient reported outcome measures (PROs). SDM involves a collaborative process of reaching a treatment decision based on patients’ values, preferences, and treatment goals, requiring active involvement from both patients and clinicians [3]. SDM can be supported by DAs, instruments that help patients understand their options, clarify their preferences, and become an informed participant [4, 5]. PROs are integral to assessing outcomes for functional urologic conditions like OAB as they are reports of health status provided by the patient without clinical interpretation [6]. PROs have been shown to improve patient outcomes including improving quality of life and decreasing symptom burden [7].

In this review, we discuss the evidenced-based management of OAB, define SDM in this context, explore its current use, and evaluate the availability of decision aids that support SDM for OAB. Finally, we discuss the integration of DAs and PROs into guideline concordant OAB care. The overarching goal is to synthesize the current literature as it relates to SDM and PROs and identify gaps to guide future research in OAB care.

## Methods

We performed a systematic review for peer-reviewed articles published between 2022 and 2025 in English, using the following search terms: “overactive bladder” AND “urgency incontinence” AND “shared decision-making” OR “decision-aid”. The search was initiated and completed on March 29, 2025 and was performed using the HOLLIS Harvard Library Online Catalog and additional hand searching was performed in Google Scholar. We included peer-reviewed articles if they addressed shared decision-making, decision aids, or patient-reported outcomes in the context of OAB care. We included randomized trials, pilot and feasibility studies, health services or observational studies, and studies that developed or implemented prediction models in the decision-making process. We excluded studies that primarily focused on pediatric populations, and phase 1 and phase 2 clinical trials, as well as reviews, meta-analyses, economic analyses, and thesis or dissertations. One co-author performed an initial screening of title and abstracts using Covidence software (Covidence, Veritas Health Innovation, Melbourne, Australia). Full-text articles were reviewed by two co-authors, and subsequently finalized the list of included records. Data were then extracted based on three main themes: (1) the role and impact of SDM in OAB care, (2) DAs used in SDM to promote preference elicitation, and (3) PROs used to assess the outcomes from patients’ perspective. Extracted data were synthesized in a narrative fashion. In addition, we searched for publicly available, patient facing decision aids. We searched three publicly available sources: the Patient Decision Aids Research Group at The Ottawa Hospital, Google Search, Google Scholar and ChatGPT by searching the terms “OAB Decision Aids” and “OAB Decision Aid Apps”. We also performed a search to compile currently available PROs using Google Scholar.

## Results

**Fig. 1:**
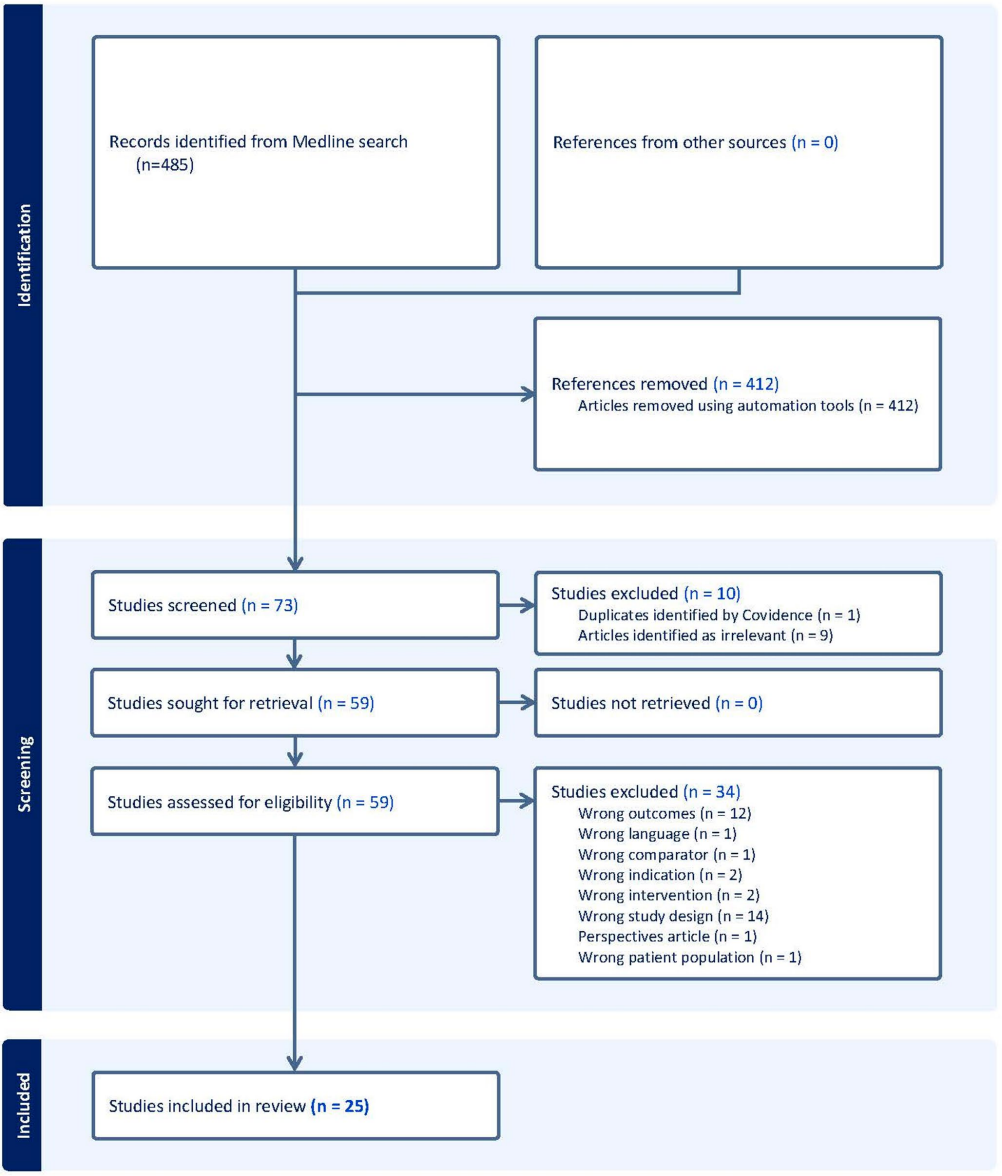
Preferred Reporting Items for Systematic Reviews and Meta-Analyses (PRISMA) flow diagram for the literature review

The literature search yielded 485 records. We excluded 412 records during abstract screening and 10 records were excluded during the abstract screening step. Fifty-nine records were sought for retrieval and 34 additional records were removed during the eligibility assessment step. Twelve records were removed for wrong outcomes (e.g., comparison of intraoperative techniques or surgery naive vs. postoperative patients etc.), 14 records for wrong study design (i.e., protocol papers, machine learning classification studies), two records for each for wrong intervention (e.g., gene therapy) or wrong indication (e.g., interstitial cystitis, vulvodenia etc.), among other reasons). Inclusion and exclusion of articles is outlined in the PRISMA flow diagram. Figure 1 The data synthesis was based on the review of 25 full-text articles Table 1.

**Table 1:** Description of Included Studies

Author (Year)	Study Type	Patient Population	Primary Outcome(s)	Shared Decision Making Implications	Patient Reported Outcome Measures Used
Chartier Kastler 2021	Prospective, multicenter, observational study	Patients receiving sacral neuromodulation (SNM) therapy for overactive bladder (OAB) or non-obstructive urinary retention based on local standard of care	Changes in voids, leaks, catheterizations/day within 12 months after InterStim implant. De novo: voids and leaks reduced. Replacement: voids and leaks reduced after 10 months	Goal attainment scale, bother scores, and QoL metrics tied to SDM Study shows symptom improvement and patient satisfaction, supporting SDM	Disease bother (Numeric Rating Scale) Goal Attainment Scale QoL (Ditrovie, Eq. 5D5L) Urinary Symptom Profile Wexner score (double incontinence patients)
Khanijow 2021	Mixed methods (Phase 1: qualitative interviews; Phase 2: prospective cohort study)	Women with OAB	Phase 1: Women with OAB dissatisfied with current treatment information Phase 2: Decision aid reduced decisional conflict and increased satisfaction with physician counseling	DAs helped women feel better informed and increased satisfaction with care	mPDA (mobile application PDA)
Ramirez Garcia 2021	Randomized control trial	Patients with refractory OAB symptoms and detrusor overactivity	Significant QoL improvement in both TTNS and PTNS groups	No SDM mentioned	QoL questionnaires (OABqSF, IQoL) Treatment benefit on symptoms questionnaire
Frankel 2021	Randomized, double-blind, placebo- and active-controlled trial	Adults with OAB (wet or dry) for at least 3 months prior to screening	At week 12, vibegron improved OABq scores (compared to placebo). Greater proportion achieved best response on PGI endpoints	Not directly mentioned, but evidence suggests SDM could optimize care by capturing the patient’s voice through OABq	OABq: coping, concern, sleep, social interaction, symptom bother PGI: severity, control, frequency, leakage, change
Chhatre 2021	Phase 1: literature review; Phase 2: cohort study	Not specified	Top attributes influencing treatment uptake: caregiver burden, impaired bladder function, social interaction constraints, treatment side effects, and outofpocket costs	Tools like OABCare can improve outcomes by enhancing communication and tailoring treatments to patient needs	OABCare instrument
Marques 2021	Pilot study	English-speaking adults aged 18–95 years with pelvic organ prolapse (POP), stress urinary incontinence (SUI), or OAB	Intervention group showed higher knowledge scores, clearer treatment preferences, and higher SURE and CollaboRATE scores	DA was efficient, increased patient awareness, and boosted confidence in treatment choices	Patient Postvisit Survey (knowledge, treatment preference, SURE scale, CollaboRATE, SDM process scale)
Mostafaei 2022	Meta-analysis	Not specified	Determined the most efficacious oral antimuscarinic or betaadrenoceptor agonist	Framework allows individualized approaches, improving outcomes based on patient preferences while minimizing adverse events	None reported
TeDorsthorst 2022	Post-hoc assessment (retrospective analysis)	Patients previously implanted with RENOVA (implantable tibial nerve stimulators)	BMI predictive of treatment success; age, sex, and prior PTNS experience were not predictive	SDM mentioned in conclusion but not discussed in detail	No PROs used
Millimet 2022	Prospective study	Patients prescribed anticholinergic medications for OAB	8438% of patients satisfied with initiative; most elected to change therapy after learning about dementia risk	Patients discussed options with physicians and decided whether to continue medication	None reported
Kraus 2022	Cross-sectional study	OAB patients and physicians treating OAB	SDM guided treatment decisions; safety and efficacy were main considerations	Increased awareness of combination therapies could improve OAB outcomes	Patient and physician surveys
Schonburg 2022	Post-hoc analysis of two large non-interventional studies	Not specified in the provided data	PPBC correlated with urgency, incontinence, micturitions, and nocturia episode frequencies (moderate strength)	PPBC captures subjective feelings, valuable for SDM conversations	PPBC (Patient Perception of Bladder Condition)
Kapur 2022	Observational study (patient education and survey)	Convenience sample of patients with OAB refractory to first- and second-line treatments, eligible for third-line therapy	Patients selected most/least attractive features of thirdline therapies PTNS attractive for low complication rate; least attractive for time commitment	None reported	Baseline symptoms assessed using UDI6 SF and IIQ7 SF
Speed 2023	Conjoint analysis	New patients with urinary incontinence	Frequent followup, catheterization risk, and device need were key decision factors for thirdline therapy	Physician input influences patient decisionmaking	Conjoint analysis survey
AalamiHarandi 2023	Retrospective review	Patients who underwent botulinum toxin A (BTX-A) injections for OAB	Retention after BTXA injection not associated with BMI	BTXA can be discussed with more patients, including those with high BMI	None reported
Welk 2020	Randomized, double-blind, sham-controlled study	Two groups: < br > 1) Women with OAB < br > 2) Patients with neurogenic disease and bladder symptoms	No significant improvement in PPBC, 24 h pad weight, voiding diary parameters, or conditionspecific PROs with TTNS	TTNS did not show significant improvement in PROs for OAB	PPBC (all patients) OABq SF (OAB patients) NBSS and QualiveenSF (neurogenic patients)
Okui 2023	Observational study	Older women who received intravesical onabotulinum toxin A injections for OAB between 2020 and 2022	Daytime frequency, frailty, and voiding inefficiency associated with PVR > 200 mL	Models can help patients feel more comfortable with treatment plans and assist in decisionmaking	None reported
Frankel 2023	Randomized controlled trial	Not specified	Vibegron significantly improved OAB symptoms and PROs compared to placebo	PROs and patientdefined meaningful changes emphasize patient involvement in care	PGIC (measures overall symptom change) Patient diaries (urination, urgency, bladder leaks)
Kessler 2024	Retrospective study	Not specified	Low regret and high satisfaction with SNM for refractory OAB after 3 years Postop complications worsened scores	SDM necessary to manage expectations and reduce decisional regret	SDSDRS (Satisfaction with Decision Scale–Decision Regret Scale)
Sitto 2024	Mixed methods	Not specified	Patients prioritized treatment invasiveness, efficacy, and safety when selecting OAB therapy	Aligning patient and physician priorities can improve SDM outcomes	LURN SI 10 (screening)
Werneburg 2024	Prediction modeling	Not specified	Neural network outperformed human experts in predicting OBTXA outcomes for OAB	None reported	None reported
Gaddam 2025	Insurance coverage analysis	US insurers	Oxybutynin IR had the best coverage score; trospium ER had the worst. Preferred medications had worse coverage than nonpreferred	Implied that patients and physicians discussed beta3 agonists, weighing costs, side effects, and other factors	None reported

## Discussion

**Fig. 2:**
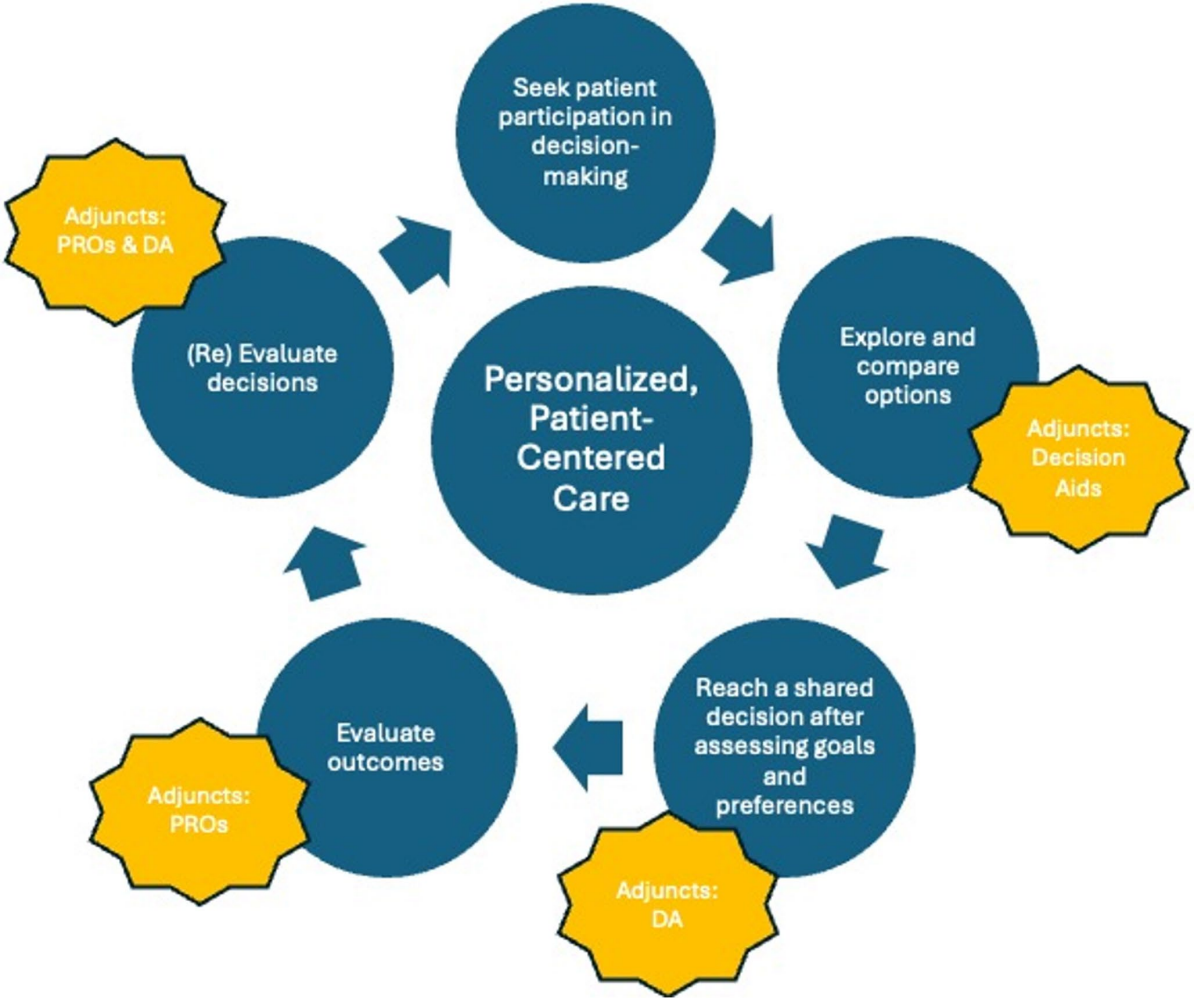
Conceptual Model of the Integration of Patient Reported Outcomes (PROs) and Decision Aids (DAs) to facilitate Shared Decision-Making

SDM was discussed in 14 of the 25 full-text articles, DA was discussed in 5 and PROs were used in 10 studies. Table 1 Our narrative review revealed that PROs and DAs support the key elements of the SDM process as depicted in Fig. 2 and outlined below.

### Evidence Based Management of OAB

Specific diagnosis and definition of symptoms is necessary for targeted, patient-centered care for people with bothersome lower urinary tract symptoms (LUTS). OAB is a syndrome with symptoms that fall within the spectrum of LUTS. The International Continence Society (ICS) defines OAB as a syndrome characterized by “urinary urgency, usually accompanied by frequency and nocturia, with or without urgency urinary incontinence, in the absence of urinary tract infection (UTI) or other obvious pathology” [8, 9]. By definition, OAB is a heterogenous syndrome with a variety of presentations, which makes patient centered care and precision treatments challenging to obtain. Recently research collaboratives such as Symptoms of Lower Urinary Tract Dysfunction Research Network (LURN) have focused on studying individual symptoms, such as urinary urgency and urgency urinary incontinence, key symptoms of OAB, to better understand and treat people with these symptoms [10]. Despite these efforts, the majority of data and clinical applications of treatment for urinary urgency and urgency urinary incontinence use the broader term OAB which we use in this review, despite acknowledging the term as a barrier to patient-centered care.

The evidence based management of overactive bladder is extensively outlined in the 2024 AUA-SUFU Guideline on the Diagnosis and Treatment of Idiopathic OAB as well as The 2025 European Association of Urology (EAU) Guidelines on Management of Non-Neurogenic Female Lower Urinary Tract Symptoms [11, 12]. In each guideline management begins with diagnosis based on history and physical exam, including obtaining PROs (symptom questionnaires) and voiding diaries. Basic evaluation of people with symptoms suggestive of OAB includes obtaining a post void residual and urinalysis (to exclude other causes of OAB-type symptoms) and while adjunct testing can be performed in the context of diagnostic uncertainty, the diagnosis of OAB does not rely on advanced testing.

The AUA/SUFU guideline offers five categories of interventions for bothersome OAB symptoms: non-invasive therapies, pharmacotherapy, minimally invasive procedures, invasive therapies and indwelling catheters [12]. Non-invasive therapies include continence management devices (pads, barrier creams), behavioral therapy, bladder training, pelvic floor muscle training, magnetic stimulation, transcutaneous tibial nerve stimulation [12]. Pharmacotherapies specific for OAB include antimuscarinics and beta-3 agonists [12].Minimally invasive therapies include sacral neuromodulation, intradetrusor botulinum toxin injection, percutaneous tibial nerve stimulation, and implantable tibial nerve stimulation [12]. Invasive therapies include augmentation cystoplasty or urinary diversion (cystectomy with ileal conduit). Indwelling catheters are only recommended when OAB therapies are contraindicated, ineffective or no longer desired, due to the risk of potential harm from chronic indwelling catheters [12].

Both the AUA/SUFU and the EAU guidelines promote the use of shared decision-making to facilitate all phases of treatment for OAB symptoms [11, 12]. While the EAU guideline maintains verbiage referring to step-therapy, the AUA/SUFU guideline clearly outlines that to date, there is no significant evidence to support or refute step-therapy which is the stepwise progression from non-invasive to pharmacological, procedural and finally major surgical interventions [11, 12]. This conclusion places even more emphasis on the ability for clinicians to understand and practice shared decision-making in the context of OAB management [12].

### Shared Decision-Making in OAB Care

Fourteen of the 25 articles we identified had implications for SDM in OAB. Table 1 To review, SDM is a process where clinicians and informed patients review medical evidence and balance this with patients’ preferences and values to reach a medical decision [3]. SDM was first introduced in 1982 and since that time has been increasingly recognized as the gold standard clinical practice to deliver high quality, personalized and patient centered care [13, 14]. SDM is recommended in both the AUA/SUFU and the EAU guidelines related to OAB care and to facilitate this both the AUA and the EAU offer guidance on the implementation of SDM in urologic care [11, 12]. The AUA released a white paper on the implementation of SDM in 2015 with a 2022 update and the AUA/SUFU Guidelines specifically detail the process of SDM in OAB [12, 15]. While many paradigms for implementing SDM exist, the essential components include engaging patients’ in the process, exploring treatment options including the potential harms, benefits and alternatives, assessing an informed patients’ preferences and values, arriving at a decision and coordinating follow-up for the decision [15, 16]. Similarly, the EAU offers webinars on “Data-driven SDM in the management of incontinence” and “Comparison of OAB treatments and SDM.” [17, 18].

Current literature identified in this review [5, 19–21] offer new insights into the complexities of SDM in OAB care. Table 1 Kraus et al. (2022) surveyed 200 patients with OAB as well as physicians (*n* = 50), and found that the majority of patients (87%) reported being involved in OAB treatment decisions [21]. However less than half of patients reported being involved in decisions to change, continue or stop treatments or to add a treatment [21]. Sitto et al. (2024) conducted a mixed-methods study evaluating patient and physician decision making in OAB [19]. The authors identified four key themes in the decision making dynamics: frustration with inaccessibility of OAB treatments, discordant perception of patient education, diverging acceptability of expected outcomes and lack of insight into the other parties’ decisional priorities and control preferences [19]. Both studies found that physician recommendation, efficacy and side effects were the most common factors patients considered when making treatment decisions. Paradoxically, the notion that decisional priorities diverge between patients and physicians was found in three studies, although two found physicians to prioritize safety over efficacy [19, 21], while one reported the opposite [20]. The paradox that physician recommendation is a key decisional component for patients yet that physicians’ priorities may differ widely from patients’ preferences serves as a striking reminder of why transparent SDM is needed for OAB care. Kessler reported high satisfaction and low decisional regret among patients who underwent SNM therapy, however, complications from the procedure negatively impacted patient satisfaction [26]. 

### The Use and Availability of Decision-Aids for SDM in OAB Care

The use of decision aids to elicit patient preferences and to support shared decision-making was identified in 5 articles included in this review [19, 22–25]. Table 1 Khanijow and Marques found that DAs improved patient knowledge, reduced decisional conflict, and increased satisfaction with care [22, 24]. In these studies, DAs supported patients to make informed decisions that aligned with their treatment goals and preferences.

### Availability of Decision Aids

Despite being useful instruments to elicit patient preferences, few publicly available DAs for OAB exist. Some DAs are publicly available and can be found online, while others are proprietary and require patients or clinicians to purchase access. In recent years, large language models (LLM) have emerged as tools to search for health information. LLMs provide information on the availability of DAs with a summary of features and benefits. However, the onus is on the user to seek that information and have the tools to access the decision aid, even if publicly available. See further discussion below in section Artificial Intelligence in ShareD decision Making for OAB care.

The academic literature finds three peer reviewed OAB decision aids: OABCare, which is a web based instrument that utilizes discrete choice experiments [25], *Streamlined* a mobile application [22], and a paper based decisional grid for minimally invasive therapies [24]. None of these tools are publicly available, though the Marques grid is available within the original publication [24]. Publicly available tools that have not been peer reviewed are available. Among these are the The University of Michigan Medicine decision aid tool for OAB [27], The World Federation of Incontinence and Pelvic Problems (WFIPP) decision aid [28], and the The SUFU OAB Clinical Care Pathway [29]. The SUFU OAB Clinical Care Pathway included a ‘My Bladder’ app that was designed to help keep patients engaged with their treatments, though this is not currently available for download. While there are apps available for digital prescription therapeutics targeting behavioral therapy, these do not include DAs [30, 31]. In addition to direct-to-consumer products, DA platforms are increasingly integrated into electronic medical records and available through health insurance companies, For example, WiserCare, a digital health company, provides a platform for patient decision support tools [32].

### Patient Reported Outcome Measures To Augment SDM in OAB Care

Shared decision-making in OAB care can be augmented by the systematic integration of patient-reported outcomes (PROs). The AUA/SUFU guidelines on OAB state that “Clinicians may obtain a symptom questionnaire and/or a voiding diary in patients with symptoms suggestive of OAB to assist in the diagnosis of OAB, exclude other disorders, ascertain the degree of bother, and/or evaluate treatment response [12]. PROs are reports of health status provided by the patient without clinical interpretation and are collected using validated instruments that have undergone usability and psychometric testing [6]. PROs have been proposed as effective instruments to advance patient-centered care as PROs capture how a patient feels, functions, and other quality of life items. In many health conditions including oncology, PROs have been shown to improve patient outcomes including improving the quality of life and decreasing health care utilization [33, 34]. Individuals living with OAB cycle through multiple lines of therapy and experience urologic symptoms that interfere with daily life including emotional and social wellbeing. 

 We identified nine studies using or discussing PROs for overactive bladder. Table 2 Many of the these studies compared the clinical efficacy of interventions (Chartier-Kastler et al. 2023; Ramírez-García et al. 2020; Welk and McKibbon 2020; Aalami Harandi et al. 2023; Okui et al. 2023; Frankel et al. 2022). The importance of PROs for OAB is underscored by the large number of PROs that have been developed to capture OAB symptoms [35–43].

**Table 2:** Patient Reported Outcome Measures in OAB Care

Patient Reported Outcome Measure	Year	Purpose	Items, Subscales or Domains	Dimensions or Factors	Scoring	Citation
Urinary Distress Inventory (UDI)	1994	Assesses the distress/bother caused by lower urinary tract syndromes (LUTS)	Short form 6 items Long form 19 items	Urinary frequency, urgency, leakage, voiding difficulties, pain/discomfort	Average scores calculated, then multiplied by 33.3 to transform into a 0 to 100 scale Higher scores indicate a greater distress	Shumaker, S. A., Wyman, J. F., Uebersax, J. S., McClish, D., Fantl, J. A., & Continence Program in Women (CPW) Research Group. (1994). Health-related quality of life measures for women with urinary incontinence: the Incontinence Impact Questionnaire and the Urogenital Distress Inventory. Quality of life Research, 3(5), 291–306.
Incontinence Impact Questionnaire (IIQ-7)	1995	Assess the impact of urinary incontinence on quality of life	Short form 7 items Long form 30 items	Impact on physical activity (household chores), travel, social relationships, and emotional health	Average scores calculated, then multiplied by 33.3 to transform into a scale of 0 to 100 Higher scores indicate a greater impact of incontinence	Uebersax JS, Wyman FF, Shumaker SA, et al. Short forms to assess life quality and symptom distress for urinary incontinence in women: the incontinence impact questionnaire and urogenital distress inventory. Neurourol Urodyn 1995; 14: 131.
King’s Health Questionnaire (KHQ)	1997	Assess the impact of urinary incontinence on quality of life	21- items 9 domains	General health perceptions, impact of incontinence on life, role limitations, physical limitations, social limitations, personal relationships, sleep or energy, emotions, and incontinence severity	Liker scale item scores converted to a linear 0 to 100 scale. Incontinence severity scored on a 0 to 30 with, 0 being the best outcome Higher scores indicate worse outcomes	Kelleher, C. J., Cardozo, L. D., Khullar, V., & Salvatore, S. (1997). A new questionnaire to assess the quality of life of urinary incontinent women. BJOG: An International Journal of Obstetrics & Gynaecology, 104(12), 1374–1379.
OAB-q	2002	Assesses patient-reported symptoms of OAB and its impact on health-related quality of life	33-items, including: Symptom bother subscale (7 items) HRQoL subscale (25 items)	Frequency, urgency, nocturia, and incontinence symptoms HRQoL: coping behaviors, concern/worry, sleep and social interaction	Summed-up scores for both domains are transformed into a linear 0 to 100 scale. Higher symptom bother scores indicate greater symptom burden. Higher QOL scores indicate better QOL	K. Coyne, D. Revicki, Hunt, T., R. Corey, Stewart, W., J. Bentkover, H. Kurth, & Abrams, P. (2002). Psychometric Validation of an Overactive Bladder Symptom and Health-Related Quality of Life Questionnaire: The OAB-q. Quality of Life Research, 11(6), 563–574. 10.1023/a:1016370925601
International Consultation on Incontinence Questionnaire - Overactive Bladder (ICIQ-OAB)	2004	Assesses urinary incontinence and its impact on quality of life	3 scored items and 1 unscored self-diagnostic item	Frequency, nocturia, urgency, and urge urinary incontinence	A score range of 0–16 with greater values indicate increased incontinence severity	Avery K, Donovan J, Peters TJ, Shaw C, Gotoh M, Abrams P. ICIQ: a brief and robust measure for evaluating the symptoms and impact of urinary incontinence. Neurourol Urodyn. 2004;23(4):322 − 30. doi: 10.1002/nau.20041. PMID: 15,227,649.
Patient Perception of Bladder Conditions (PPBC)	2006	Assess patient’s perception of their urinary problems	1-item	Bladder condition rated on a 6-point Likert-type scale	Lower scores indicate a better perception of bladder condition	Coyne, K. S., Matza, L. S., Kopp, Z., & Abrams, P. (2006). The validation of the patient perception of bladder condition (PPBC): a single-item global measure for patients with overactive bladder. European urology, 49(6), 1079–1086.
OAB Symptom Score (OAB-SS)	2006	A single symptom score using a self-reported questionnaire to quantify OAB symptoms	4 items ranging from 0–5	frequency, nocturia, urgency, urgency incontinence	Total score is 15, higher score indicates more severe symptoms	Homma Y, Yoshida M, Seki N, et al. Symptom assessment tool for overactive bladder syndrome–overactive bladder symptom score. Urology. 2006;68(2):318–323. doi:10.1016/j.urology.2006.02.042
Michigan Incontinence Symptom Index	2014	Validated score developed to discern between type of incontinence and the severity and bother caused by urinary incontinence	10 items with 2 domains Total Domain (stress urinary incontinence and urgency urinary incontinence and pad use) and Bother Domain.	Stress Incontinence, Urgency Incontinence, Frequency, nocturia, pad use, Bother	The Total M-ISI Domain consists of 3 subdomains (items 1–3 for SUI, items 4–6 for UUI, and items 7–8 for Pad Use. he Total M-ISI Domain ranges from scores of 0 to 32, the Bother Domain ranges from scores of 0 to 8, the SUI and UUI Subdomains range from scores of 0 to 12, and the PU Subdomain ranges from scores of 0–8	Suskind AM, Dunn RL, Morgan DM, DeLancey JOL, McGuire EJ, Wei JT. The Michigan Incontinence Symptom Index (M-ISI): a clinical measure for type, severity, and bother related to urinary incontinence. Neurourol Urodyn. 2014;33(7):1128–1134. doi:10.1002/nau.22468
LURN SI-29 or the LURN SI-10	2019	Assess urinary symptoms in men and women with lower urinary tract symptoms including OAB	Long form 29 items (27 items for both sexes, 2 sex-specific items) Short form 10 items	Urinary urgency, incontinence, voiding difficulty, nocturia, pain, frequency and post-micturition symptoms	The subscale and total scores for LURN SI-29 are normalized to 0–100 Higher scores indicate greater severity of LUTS	Cella, D., Smith, (A) R., Griffith, J. W., Flynn, K. E., Bradley, C. S., Gillespie, (B) W., … & LURN Study Group. (2019). A new outcome measure for LUTS: symptoms of lower urinary tract dysfunction research network symptom Index-29 (LURN SI‐29) questionnaire. Neurourology and urodynamics, 38(6), 1751–1759.

PROs have been integrated into major EHRs as the evidence to support their use in routine practice is increasing [44]. These EHR integrated PROs allow clinicians to remotely monitor patients’ symptoms between clinic visits. Monitoring of symptoms, function status, and HRQOL in between clinic visits has been shown to be more robust than in-clinic symptom assessments because in-clinic assessments may be affected by recall or recency biases [45]. For example, patients with memory loss, cognitive decline, or early-onset dementia may not accurately remember their symptoms between clinic visits. Therefore, remote symptom monitoring through PROs provide a valuable way to collect symptoms to help inform the treatment decision-making process. In OAB, Digital Clinical Care Companions, embedded within the EHRs, leverage PROs for asynchronous care for OAB [46].

Treatment decisions based on PRO data are more responsive to patients’ changes in conditions or health status, resulting in a higher quality SDM process. Systematic monitoring of PROs allows clinicians to assess symptoms over time and measure changes in symptom manifestation including frequency, severity, and co-occurrences, thereby providing actionable information to help support the decision-making process. Treatment decisions that reflect changes in symptoms and functional statuses are more responsive to patients' conditions. Further, incorporating longitudinal PRO data enables higher quality decision-making that is more responsive or reflective of not only the patients' values, preferences, and treatment goals but also their current health status.

### Artificial Intelligence in Shared Decision Making for OAB Care

Artificial intelligence (AI) is emerging as a valuable tool to enhance shared decision-making for OAB. Machine learning provides enables the development of clinical prediction tools to help provide individualized outcome estimates to guide shared decision-making. In addition, Large Language Models (LLMs) can be used for data extraction, clinical recommendations, and as patient-facing chatbots, thereby supporting both physicians and patients [47]. LLMs are especially attractive in the context of SDM because they could provide easily accessible, balanced information to clinicians and patients.

Clinical prediction models using machine learning have been developed to support treatment decision-making in OAB. Several models have been trained and developed in OAB, however, two recent examples are noteworthy. A random forest model was found to be 80.3% accurate at predicting the failure of anticholinergic treatment [48]. In another example, Werneburg found that a neural network model was better than humans in predicting OAB outcomes for intravesical botulinum toxin injections [48, 49]. These models may provide useful supporting information that can be factored into SDM discussions, thus enhancing the shared decision-making process and improving patient outcomes. However, the use of machine learning algorithms for clinical prediction and decision-making is not without challenges. Most importantly, prediction models are susceptible to over-fitting, biases introduced in the model training process, concerns about external validation as well as the need to monitor model performance over time and updating or retraining models as new data become available [50]. Furthermore, studies of urologists have found that only 30% self-reported regular use of validated prediction tools [51]. 

LLM models such as Med-PaLM from Google are specific to medical and clinical applications. However, the most accessible and widely used models are ChatGPTs from OpenAI, Llama from Meta, and Gemini from Google [52]. Zhou et al. found that ChatGPT can provide responses that are in line with the highest-quality evidence, although it lacks clinical experience and judgment [53]. The use of these LLMs comes with risks; for example, in a study evaluating the quality of information for urologic patients, Cocci et al. found that only 52% of ChatGPT responses to questions on likely diagnosis, suggested examinations, and treatment options were deemed appropriate by a board-certified urologist who was blinded to the LLM's responses [54]. This suggests that open LLM models may need further refining before use in SDM.

## Conclusions

This review found SDM enhanced patient engagement, improved satisfaction with care, and reduced decisional conflict. DAs served as preference elicitation tools that facilitated the SDM process between patients and clinicians, although few are publicly available. SDM enabled providers to align management strategies with patient values, preferences, and goals. Patients value safety, efficacy, and treatment costs among other attributes. Further, involving patients in the treatment decision-making process led to better alignment between prescribed treatments and patients' goals, which contributed to higher satisfaction with treatment. Several PROs are available and are increasingly being integrated into electronic health records and digital OAB care companions. The use of AI applications including machine learning and large language models, is emerging as a valuable tool to support patient education and the treatment decision-making process. However, risks and limitations of these tools are real and the potential to cause harm is greater if the information or tools are not validated by experts.

## Key References

- Ippolito GM, Reines K, Meeks WD, et al. Perceived vs. actual shared decision-making behavior among urologists: A Convergent, Parallel, Mixed-Methods Study of Self-Reported Practice. Urology. Published online November 21, 2023. https://doi.org/10.1016/j.urology.2023.10.026.
  - ⚬ A key study that found urologists often overestimate how much SDM they practice, revealing a perception-practice gap and highlighting the need for objective assessments, ongoing training, and support to foster more SDM in routine practice.
- Cameron AP, Chung DE, Dielubanza EJ, et al. The AUA/SUFU guideline on the diagnosis and treatment of idiopathic overactive bladder. Neurourol Urodyn. Published online July 15, 2024. https://doi.org/10.1002/nau.25532.
  - ⚬ AUA/SUFU guideline on idiopathic OAB that emphasizes a patient-centered, symptom-based treatment approach. The recent guideline emphasizes SDM and discourages rigid stepwise escalations so treatment can be personalized to patients’ history, treatment goals, and comorbidities.

## Data Availability

No datasets were generated or analysed during the current study.
